# Mandating the Use of Proximity Tracking Apps During Coronavirus Disease 2019: Ethical Justifications

**DOI:** 10.3389/fmed.2020.590265

**Published:** 2020-12-02

**Authors:** Riya Dave, Rashmi Gupta

**Affiliations:** Cognitive and Behavioural Neuroscience Laboratory, Department of Humanities and Social Sciences, Indian Institute of Technology Bombay, Mumbai, India

**Keywords:** ethical framework, digital health, COVID-19, ethics, contact tracing

## Abstract

The rise of the coronavirus disease 2019 (COVID-19) in a digital world has expectedly called upon technologies, such as wearables and mobile devices, to work in conjunction with public health interventions to tackle the pandemic. One significant example of this integration is the deployment of proximity tracking apps on smartphones to enhance traditional contact tracing methods. Many countries have adopted proximity tracking apps; however, there is a large degree of global differentiation in the voluntariness of the apps. Further, the concept of a mandatory policy—forcing individuals to use the apps—has been met with ethical concerns (e.g., privacy and liberty). While ethical considerations surrounding deployment have been put forth, such as by the World Health Organization, ethical justifications for a mandatory policy are lacking. Here, we use the Faden–Shebaya framework, which was formed to justify public health interventions, to determine if the compulsory use of proximity tracking apps is ethically appropriate. We show that while theoretically justified, due to the current state of proximity tracking applications and societal factors, it is difficult to defend a mandatory policy in practice.

## Introduction

The rise of coronavirus disease 2019 (COVID-19) during a digital technology boom has led the world to integrate various technologies with health strategies to curb the pandemic. Amongst such integrations, the use of smartphones in conjunction with traditional contact tracing methods has been proposed as a key way to augment public health surveillance (1–3). To do so, digital proximity tracking apps—location tracking applications downloaded onto smartphones—have been globally issued. Proximity tracking apps measure the signal strength between smartphones to determine whether two devices were close enough for a long-enough duration for there to have been virus transmission. If an individual is infected, those within proximity of the infected individual will be notified. Appropriate next steps to reduce health risks are then given to the suspected individual (1–5).

The proposed benefits of proximity tracking apps have encouraged many countries to design and deploy such tools quickly (1). However, there is large global differentiation in their voluntariness, as some countries mandate that individuals download the app (3, 5, 6). While app usership has proposed benefits, mandating their use has led to ethical concerns over the infringement of individual rights (liberty and privacy). Noting the tradeoff, the World Health Organization (WHO) has outlined ethical suggestions for how governments and private institutions could design and deploy proximity tracking apps (1). Recent studies have also put forth their ethical considerations as frameworks for app implementation (3, 6, 7).

Although considerations and suggestions have been put forth, ethical justifications for a compulsory intervention are lacking. Is it ethically appropriate to mandate the use of proximity tracking apps despite violations to individual rights? Given the global differentiation in the voluntariness of proximity tracking apps and their proposed benefits to public health (8), we believe that an investigation of the ethical appropriateness of mandating their use is highly necessary.

## Frameworks for the Ethical Justification of Public Health Interventions

To justify the mandatory implementation of digital proximity tracking apps, we can turn to frameworks that determine the ethical appropriateness of public health interventions. Amongst frameworks, there is differentiation in the themes emphasized: For example, Kass (9) and Childress et al. (10) highlight the intervention efficacy plays in justifying public health intervention (11), while Upshur (11) and Faden and Shebaya (12) believe the straightforward application of the principles of biomedical ethics—autonomy, beneficence, nonmaleficence, and justice—is too limited in scope.

The “Faden–Shebaya framework” further differs from other frameworks in that it argues against frameworks that provide broad, moral warrants, such as “to maximize public good” or “advance social justice” (12). The “Faden–Shebaya framework” does not deny that the underlying tension of a health policy surrounds hurting individual rights, but they do not disproportionately weigh “human flourishing” to justify any violations to autonomy (12, 13). In other words, the framework incorporates factors, as discussed below, in addition to public and individual health benefits into the core of their ethical debate. Given the focus of the framework across channels and the emphasis on more than the scientific efficacy of the policy, we use the “Faden–Shebaya framework” to justify whether mandating the use of proximity tracking apps is ethically appropriate. To do so, we critically analyzed four justifications they have put forth: (1) collective action, (2) overall health benefit, (3) distribution of burdens, and (4) harm to others (Mill's harm principle) (12). Before delving into the framework, we first discuss the individual rights that may be violated. We then explain the justifications of the framework in detail. Finally, an ethical analysis that incorporates these elements is provided.

## Implications of a Mandatory Policy on Individual Rights

In the event of any mandatory intervention, three individual rights are known to be violated: liberty, privacy, and informed consent (13).

With this intervention, liberty is violated as individuals do not have the ability to reject the policy. Privacy regards data misuse, such as the transfer of personal information outside the defined goals of the app, imperfect anonymization of the data, or security loopholes in the app that put individual data at risk (14). Without informed consent, the agent in charge of the dataset could collect and repurpose data from the app without the user's knowledge (3, 6, 14). Questions then surround: how long will the agent hold the data? When will it be deleted? The WHO suggests that data be deleted from proximity tracking apps after the pandemic subsides. Given the large uncertainty of when that could be, if individuals are forced to abide by such a compulsory policy, they lose their ability to not only consent to how data is collected and where it may go but also the duration of that collection (1, 3).

Further, the data collected should be anonymized and typically is even in countries that have mandatory interventions, such as in India. However, recent studies have shown that machine learning can, somewhat easily, re-identify data, which puts an individual's right to privacy on a tenuous support (15). Lastly, a mandatory policy would force individuals to face the consequences of any product malfunctions in safeguarding data. As case examples, countries such as South Korea and Qatar have been scrutinized due to security issues found in their tracking apps that put their population at risk (16).

## The Faden–Shebaya Framework

### Collective Action

Collective action is the idea that if an individual or a large group of individuals refute a public health regulation on the grounds that it does not directly benefit them or align with their beliefs, the consequences extend to society. A classic example of this concept is an outbreak of measles that resulted from under-vaccination of children by parents (17). In other words, collective action asserts that in order for an intervention to be successful, participation must encompass the entire society, as without full cooperation, neither the individual nor the society can reap the benefits of the intervention. Collective action, therefore, sets the grounds for supporting a mandatory health intervention (12, 13). Without collective action, there is also a high possibility that the “free-rider” problem will rise, where those individuals who are omitted from the intervention still gain some benefits (13).

### Fairness in the Distribution of Burden

Public health “burdens” are understood as both the burdens of the illness and the burdens of the intervention itself (13). On the grounds of fairness in the distribution of burdens, individuals may be asked to bear public health burdens that do not directly benefit them in an attempt to make the disease burdens more equitable. For instance, between 1962 and 1994 in Japan, children were also asked to be vaccinated against seasonal influenza to protect the elderly (who were harshly impacted by the illness) (12, 13).

### Overall Benefit to Society

Proponents of intrusive public health interventions often argue that such interventions are justified because of the overall benefits to society (12, 13, 17). For example, by mandating that everyone get a vaccine or requiring an HIV positive patient to disclose sensitive information on previous sexual partners, it is believed that society will benefit as a whole (13). However, in order to reap any benefits, the intervention must be effective in producing an advantageous outcome.

### Harm to Others (Mill's Harm Principle)

According to Faden and Shebaya, the “harm principle” is often viewed as the most compelling justification for public health policies that interfere with individual liberty (12). Mill's harm principle argues that harm should be prevented from occurring to others. This logic has been used to justify drastic actions such as quarantines and other compulsory treatment for highly infectious diseases (13).

## Ethical Analysis of Mandatory Policy

We segmented the analysis into two parts. The first is a theoretical justification of the intervention, followed by a review of the application of the policy in practice. We argue that while the policy may be theoretically justified, in practice, it does not hold.

### Theoretical Justification of the Intervention

The WHO states that at least 60% of a country's population needs to use the app in order to stop transmission and contain the virus (1, 4). Thus, not downloading the app will do harm to society, and by Mill's principle, the harm done to the public (contracting the virus) could have been prevented through app usage. Thus, a mandatory policy would ensure that a majority uses the app, minimizing any physical harm from illness.

The mandatory policy could be further justified on the grounds of collective action and overall health benefit, as a negative consequence of not having full participation is a suboptimal, or ineffective, contact tracing app (1). Thus, to prevent “free riders” and reap the overall health benefit, a mandatory policy would be theoretically appropriate.

Lastly, on the grounds of fairness in the distribution of burdens, a policy mandating the use of proximity tracking apps may be justified here. In general, the disease places a greater burden on the elderly: those above the age of 65 have an 80% mortality rate from the virus (COVID-19), making them an age group that is hit disproportionally more than younger generations by the virus (18). This group also has the lowest smartphone penetration rate than other generations (19). With a mandatory policy, younger generations would take on the burden of complying with the intervention (sacrificing their autonomy) in order to be in fair alignment with the disease burden placed on the elderly.

### Justifications in Practice

However, even with a mandatory policy, there are uncertainties, product concerns, and societal parameters that limit the theoretical implementation of a mandatory policy.

The impact will only go as far as the number of people that own smartphones with GPS/Bluetooth capabilities for tracking. In other words, while the policy may be theoretically justified, it is not in practice because it may be inherently impossible for everyone—or the majority—of a country to meet the user threshold suggested for app efficacy. For example, in India, 26% of the population own smartphones. While benefits may still be reaped at this percentage for that group that participates as well as others (4), this would contribute to the “free-rider” problem and contribute to skewed data (13). In addition, if this percentage lies largely within wealthier classes, then the data yielded from the app that are analyzed for alleviation purposes, such as resource allocation, would inaccurately paint an understanding of virus spread—or “hotspots” (5, 6). On the other end, this 26% would give away their autonomy but not gain a true indication of when they may be around infected people, which violates the principle of reciprocity (11, 12). Further, even if a country had the capacity to reach the needed threshold, we must also take into account cultural differentiation. From a draconian government that may lead citizens to more readily accept a compulsory policy to a prevailing religious view, such as to limit the use of technology, that may hinder acceptance of that same policy, there is no guarantee that citizens of a certain country will, in fact, follow without resistance or protest. Thus, while cooperation from all individuals in the society would eliminate any discrimination or data bias, the app's effectiveness only holds true if there is an even distribution of smartphones, a willingness to accept the policy, and a high smartphone penetration rate.

It is true that app usage is proposed to hinder virus transmission and thus control the virus spread (achieve overall health benefit). However, these benefits are contingent on a baseline requirement: that the policy proves effective in producing societal benefits. In its current state, those dependencies are not guaranteed.

The dependencies can be divided into two categories: (1) the technology itself and (2) societal parameters. With respect to the technology itself, currently, we cannot guarantee that the proximity apps will be effective and accurate in augmenting contact tracing. There is little scientific evidence of their efficacy to date (1, 3). As a result, proximity tracking apps are being deployed in many countries after few, if any, pilot studies or risk assessments published (20). In the absence of official validation tests and protocols, there can be no indicator of accuracy and effectiveness (3, 20). Other limitations include an inability to account for factors that are specific to the environment, such as wind direction or the presence of ventilation (21). In addition, while GPS and Bluetooth technologies can determine proximity, one loophole includes barriers between people, such as walls or windows, that will not automatically be factored into risk profiles. Moreover, individuals may be spatially distanced but occupy the same GPS coordinate, leading to false positives for notifications (3, 21).

Further, there are various societal parameters necessary to ensure app success, such as a high smartphone penetration rate, feasibility and reliability of testing, and individual adherence to suggested protocols. As discussed previously, to reach the proposed efficacy, the country must have a majority using GPS/Bluetooth-enabled smartphone devices (3). If we take a country such as Pakistan, which has a smartphone usership of 16%, while there would still be benefits to a compulsory policy, it could be argued that societal benefit is not being maximized for all of society, and thus, the policy is not ethically appropriate by the justification of societal benefit (22).

The next hindrance to the effectiveness of a mandatory policy is the feasibility and reliability of testing. Without the ease of testing and quick testing-response rates, the app's efforts will be thwarted. Similarly, if the testing is unreliable, then the app will not present an accurate representation of the spread of the virus. According to research done by Johns Hopkins Medical School, there was a 38% chance of a false negative, which changes to 20% if an individual was tested 8 days after infection (23). Further, the policy also requires that society members adhere to suggested protocols and self-report symptoms (if applicable). If an individual receives a notification that they were in proximity with an infected individual, but do not follow requested protocols (self-quarantine, report any symptoms later or get tested), then the app's goal will not be realized, making the collection of data and forced use come at a high cost and little societal benefit (1). Thus, while the mandatory policy can be theoretically justified on the grounds that it is benefiting public health, the uncertainty that surrounds the success of the intervention and the technology in producing public good makes it difficult to defend its implementation (3).

While the physical harm to others may be minimized through app deployment, we must not omit other forms of harm that could be placed on society members as a result of a mandatory policy. Harm, such as security threats or psychological harm from being coerced into an act against will, must be weighed (13). It is evidenced through South Korea and Qatar that a rush to design the app with minimal validation tests has led to security issues (3, 15). Faulty technology is more susceptible to data breaches, which places the individuals forced to use the app at high risk of being identified (21). In its current state, it is difficult to justify the mandatory implementation of the app under the principle that it will reduce the harm done to others by protecting them, as the app's efficacy is yet questionable. Nonetheless, to determine a justification based on harm principles, all forms of negative impact must be weighed.

## Conclusion

Here, we applied the “Faden–Shebaya framework” to determine if and how the mandatory use of contact tracing apps could be ethically appropriate. We went through their framework and critically analyzed each justification for its application to the current pandemic.

Faden and Shebaya (12) state that more than one justification can and should be used when making health policy decisions (12). While the concept of equitable distribution of burden holds in theory and in practice, when weighed with evidence from the other justifications, it is difficult to defend the policy. Therefore, we argue that while the policy could theoretically be appropriate, given the current context, such as the feasibility of testing or app limitations, it is difficult to justify a mandatory policy in practice at the expense of individual rights.

To better balance theoretical and practical justifications, there are actions that those in charge of developing and deploying such apps could take. Developers could form policies, similar to a Hippocratic Oath, to ensure that the patient is always valued first and treated ethically. This would support guidelines on data use from the app, safety testing for security loopholes, and data anonymization. Those in charge of deploying the app could take the time to continuously weigh the individual risk with societal benefit to determine the worth of deploying such apps. While there is no perfect system or answer, especially given the large cultural differentiation between countries, steps can be taken to bring about an ethical justification for a country that balances the theoretical with the practical and the individual with society.

While each health intervention taken during the pandemic, from mandatory use of masks to social distancing requirements to the prohibition of gathering, can be relayed, they each warrant their own system of justifications and cannot be treated equally. Thus, further discussion of the mandatory use of contact tracing apps is critical. What this article can conclude is that a system of checks and balances is needed before any health intervention is justified.

## Data Availability Statement

The original contributions generated for the study are included in the article/supplementary material, further inquiries can be directed to the corresponding author/s.

## Author Contributions

All authors listed have made a substantial, direct and intellectual contribution to the work, and approved it for publication.

## Conflict of Interest

The authors declare that the research was conducted in the absence of any commercial or financial relationships that could be construed as a potential conflict of interest.
